# Banded Versus Non-banded Sleeve Gastrectomy: 5-Year Results of a 3-Year Randomized Controlled Trial

**DOI:** 10.1007/s11695-023-06982-9

**Published:** 2023-12-18

**Authors:** Jodok M. Fink, Marina Reutebuch, Gabriel Seifert, Claudia Laessle, Stefan Fichtner-Feigl, Goran Marjanovic, Mira Fink

**Affiliations:** Department of General and Visceral Surgery, Centre for Surgery, Centre for Obesity and Metabolic Surgery, Medical Centre, University of Freiburg, Hugstetter Strasse 55, 79106 Freiburg, Germany

**Keywords:** Bariatric surgery, Banded sleeve gastrectomy, Sleeve gastrectomy, Weight loss

## Abstract

**Purpose:**

Banded sleeve gastrectomy (BSG) has been shown to enable better weight loss than non-banded sleeve gastrectomy (SG) in retrospective analyses. These findings were supported by two randomized controlled trials (RCT). However, to date, mid-term prospective data is not available.

**Materials and Methods:**

We invited all 94 patients of an RCT comparing banded to non-banded sleeve gastrectomy at 3 years (DRKS00007729) for a 5-year follow-up visit. Eighty-two patients (BSG *n* = 42; SG *n* = 40) came for evaluation. Outcome measures were identical with the RCT to allow longitudinal comparison. Data analysis was descriptive and focused on biometric data, development of comorbidities, mid-term complications, quality of life, and type of body contouring surgery (BCS).

**Results:**

The per-protocol analysis revealed a treatment difference of 9% (CI − 1.5 to 19.6) excess weight loss (EWL). Total weight loss (TWL) was 27.4% (CI 23.5–31.3) after SG and 31.6% (CI 27.3–35.5) after BSG. Twenty percent of patients after SG and 11.9% following BSG had been converted to a gastric bypass. Type 2 diabetes went into remission in most patients (SG 66.7% vs. BSG 63.6%). Antihypertensive medication was stopped or reduced in 81.3% after SG and 80% after BSG. Reflux symptoms were similar in both groups (symptoms $\ge$ 1/ week: SG 28.2% vs. BSG 26.8%). Frequency of postprandial regurgitation was higher after BSG (SG 23% vs. BSG 59%). Forty percent of patients had undergone BCS at time of follow-up.

**Conclusion:**

Five-year weight loss after BSG was 9% EWL and 4.2% TWL higher compared to SG. The main added morbidity following BSG was postprandial regurgitation.

**Graphical abstract:**

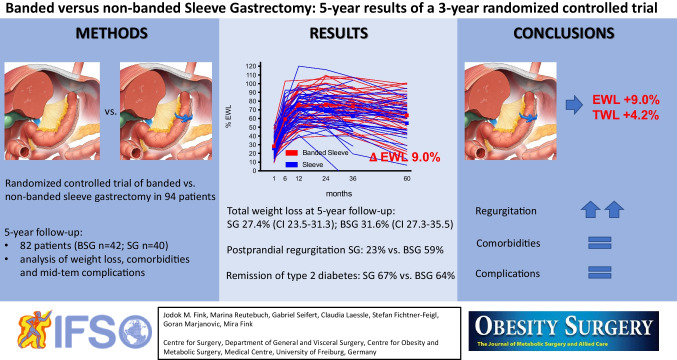

## Introduction

Sleeve gastrectomy (SG) has been shown to produce excellent mid- to long-term weight loss results [1, 2]. Although reflux symptoms, reflux esophagitis, and the development of Barrett’s esophagus pose a major concern after SG, patients overall experience a lower incidence of complications, re-interventions, and overall mortality compared to a standard Roux-en-Y gastric bypass (RYGB) [3, 4]. Yet, high volume and pooled data show superior weight loss after RYGB in relation to SG [2, 5]. In a large European patient cohort, the estimated treatment difference between the two procedures was 10.1% excess BMI loss 7 years after surgery [2]. On these grounds, the concept of improving weight loss after SG seems plausible. With this goal, our group had introduced the procedure of a primary silicone banded sleeve gastrectomy (BSG) in 2009 [6]. Compared to non-banded SG, retrospective studies so far demonstrated a significantly greater weight loss after BSG with a difference of 14% excess weight loss (EWL%) 5 years after surgery in our own series [7–9]. The major side effect of silicone ring implantation was regurgitation [7, 8]. Supporting these results, the two available RCTs comparing SG to BSG demonstrated better weight loss 3 and 4 years after BSG [10, 11]. To this date, the RCT conducted by our own group represents the largest prospective trial on banded versus non-banded sleeve gastrectomy [10]. The primary endpoint of this RCT was EWL% at 3 years, demonstrating an EWL difference of 11.6% [10]. However, prospective long-term data on BSG are not available to date.

With the aim of gathering long-term data in a prospective cohort, all patients of the initial RCT were re-examined 5–6 years after surgery primarily assessing weight loss, complications, and change in comorbidities.

## Methods

### Study Design

This study is a follow-up report of the 3-year RCT cohort comparing SG to BSG [10]. This report includes data on weight loss, resolution of comorbidities, and reflux symptoms 5–6 years after surgery. The main objectives are estimation of weight loss and weight regain in both treatment arms. Patients were followed-up from June 2021 to February 2022. This follow-up report was approved by the local ethics committee and conducted in accordance with the principles of the Declaration of Helsinki. Study design and randomization of the RCT (DRKS00007729) have been published previously [10]. All patients gave written informed consent for this follow-up evaluation.

### Participants

As this is a follow-up report on an RCT, eligibility criteria were as published for the RCT [10]. In general, eligibility criteria reflected the major indication and contraindication criteria of the German S3 guidelines [12].

### Intervention

SG and BSG were performed as described in detail in our RCT [10]. In short, sleeve formation was undertaken under perseveration of the antrum using a 35Fr. bougie. The silicone ring (MiniMizer®, Bariatric Solutions, Switzerland) was placed 4 cm below the gastroesophageal junction applying a perigastric technique. The ring was closed at a circumference of 7.5 cm and fixed to the anterior gastric wall. As patients with large (> 5 cm) hiatal hernias were excluded from the study, simultaneous hernia reconstruction was not performed.

### Outcome Measurements

All patients of the RCT cohort were invited to the institution’s outpatient clinic 5–6 years after SG or BSG. Outcome measurements at 5–6 years deliberately mirrored outcome measurements of the RCT [10]. Weight loss was recorded as %EWL and total weight loss (%TWL). Furthermore, weight regain and number of poor responders (%TWL < 20%) were assessed. Secondary bariatric operations were registered as conversions to RYGB and banded RYGB (BRYGB), or as revisions including re-sleeve gastrectomy, ring removal, or enlargement. Event and type of body contouring surgery (BCS) were recorded as arm lift, leg lift, abdominoplasty, or reconstructive breast surgery.

Type 2 diabetes (T2D) was quantified by use of oral medication, Insulin, Metformin, or GLP-1 analogue and HbA1c value. Degree of T2D remission was defined as described earlier [10]. Number of antihypertensive agents was grouped as 1–2, 3–4, or $\ge$ 5. Quality of life was evaluated using the Bariatric Analysis and Reporting Outcome System (BAROS) [13]. Patients experiencing heartburn, acidic reflux, or retrosternal pain were considered to suffer from reflux symptoms. Postprandial convulsive regurgitation of undigested food was labeled as “regurgitation.” Both symptoms were documented as either not present or as an incidence of $\ge$ 1/week, $\ge$ 1/month, or < 1/month. Reflux symptoms were additionally assessed using the Reflux Symptom Index (RSI) and GERD-HRQL score. Smokers and non-smokers were identified. Frequency of bowel movements was measured as per week. Blood serum values for Vitamin D3, B1, B12, and folic acid were determined. Deficiency was defined as serum value below ranges quoted in Table 2. Late postoperative complications were defined as major or minor following guidelines of the American Society for Metabolic and Bariatric Surgery [14].

### Statistical Analysis

All statistical analyses were conducted on a descriptive level, as this was a follow-up report of an already completed RCT and thus not powered to compare clinical outcomes differences at 5 years. Continuous variables were characterized using mean and confidence interval (CI). For categorial variables, absolute numbers or percent of patients were noted. Weight loss was the primary endpoint of the RCT and the focus of the current follow-up report. Therefore, weight loss assessment was performed as an intention-to-treat and a per-protocol analysis. For all further analyses patients were included as intention-to-treat. A linear mixed-model for repeated measures (MMRM) was used to estimate mean weight loss in both treatment groups, similar to the RCT [10]. We provide the confidence intervals of both estimates. The comparison groups were too small for a formal test due to partial loss to follow-up, and furthermore the study was not powered for this 5-year outcome comparison. We defined “patient” as a random effect, and group allocation, follow-up visit, type of surgery-by-visit interaction, baseline value of the outcome, gender, age, and presence of T2D as fixed effects. An unstructured, within-patient covariance structure was assumed.

Prism 9.4 for macOS (GraphPad Software, LLC) and SAS (SAS Institute Inc.) were used for statistical analysis.

## Results

### Patients

**Fig. 1:**
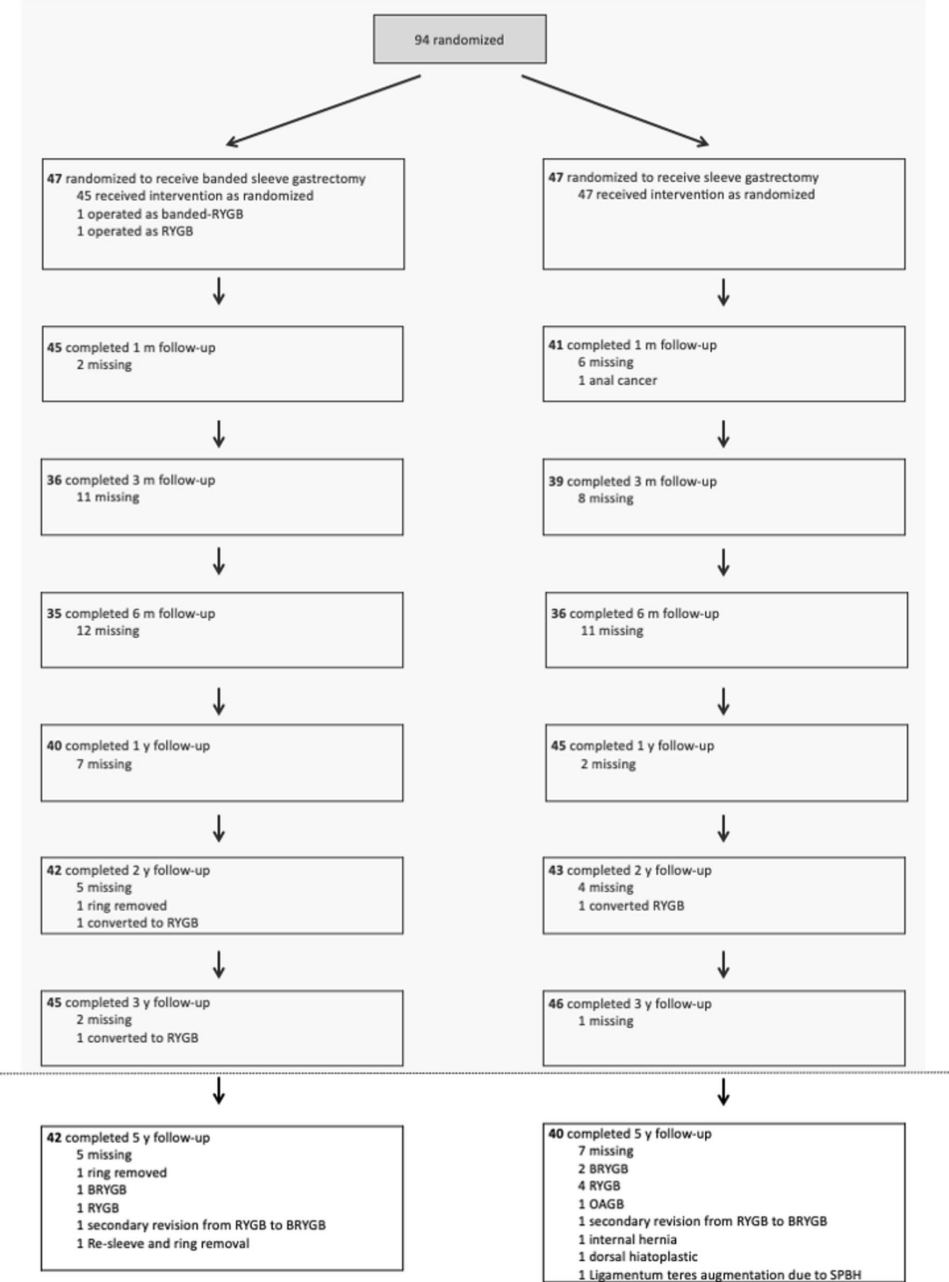
Flow chart of all patients throughout the randomized controlled trial (RCT) and current follow-up. Results of the RCT (dotted line) were published previously [10]. The chart enumerates all bariatric revisions. In total, 8 revisions were conducted in 6 patients after BSG and 12 revisions in 8 patients following SG

The patient cohort was described in detail in our RCT [10]. The 5- to 6-year follow-up was completed by 40 patients (85% of the initial study group) after SG and 42 patients (89% of the initial study group) after BSG. Figure 1 depicts the course of patients throughout the study. Twelve secondary bariatric operations (8 patients) were performed after SG, 8 (6 patients) following BSG. This resulted in different types of bariatric procedures present at the time of the 5- to 6-year follow-up in patients included in the intention-to-treat analysis. These are subdivided in Table 1. For per-protocol evaluation, 32 patients (68% of the initial study group) were included in the SG group, 35 patients (74% of the initial study group) in the BSG group.

**Table 1:** Type of bariatric procedure present in all patients included in the intention to treat analysis at 5- to 6-year follow-up

Characteristic	Banded sleeve gastrectomy (*n* = 42)	Sleeve gastrectomy (*n* = 40)
Sleeve gastrectomy	2 (4.8%)	32 (80%)
Banded sleeve gastrectomy	35 (83%)	-
Roux-en-Y gastric bypass	2 (4.8%)	4 (10%)
Banded Roux-en-Y gastric bypass	3 (7.1%)	3 (7.5%)
One-anastomosis gastric bypass	-	1 (2.5%)
Missing at follow-up (% of 47)	5 (10.6%)	7 (14.9%)

Nicotine consumption was higher following SG (SG 42.1% vs. BSG 24.4%). Stool frequency was slightly lower after BSG (BSG 5.4 (CI 4.2–6.5) vs. SG 7.0 (CI 5.8–8.3) per week). This corresponds to a higher proportion of patients experiencing 3 or less bowel movements per week in the BSG cohort (BSG 39.0% vs. SG 15.4%).

### Weight Loss

**Fig. 2:**
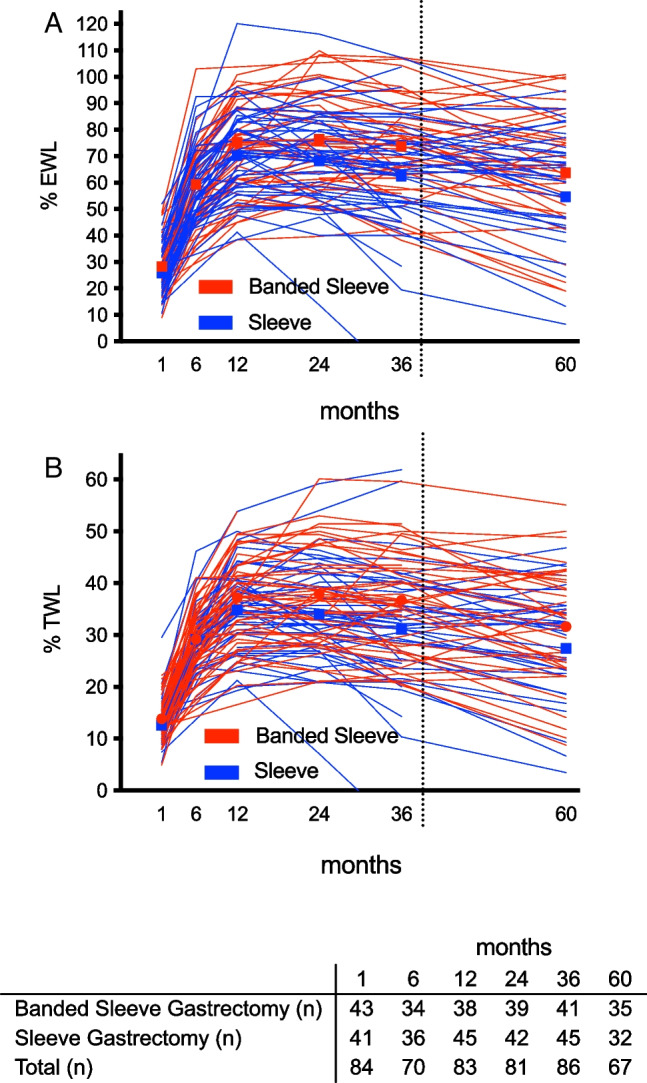
Spaghetti plot depicting excess weight loss (%EWL; Fig. 2A) and total weight loss (%TWL; Fig. 2B) after sleeve gastrectomy (SG) and banded sleeve gastrectomy (BSG). Displayed data relate to the per-protocol population. Bold lines indicate mean modelled weight loss of the mixed effects analysis. The estimated $\mathrm{\%EWL difference}$ between SG vs. BSG was 9.0%, the %TWL delta 4.2%. Data to the left of the dotted line depict the RCT results and have been published previously [10]

Estimated EWL in the per-protocol population was 54.6% (CI 47.0–62.3) after SG and 63.7% (CI 56.1–71.3) after BSG resulting in an EWL difference of 9.0% (CI − 1.5 to 19.6, Fig. 2A). The intention to treat analysis revealed an estimated treatment difference of 6.3% (CI − 4.0 to 16.7; SG 57.3% (CI 49.7–64.8) vs. BSG 63.6% (CI 56.2–71.0)). TWL amounted to 27.4% (CI 23.5–31.3) for SG and 31.6% (CI 27.3–35.5) for BSG in a per-protocol analysis (Fig. 2B). Estimated TWL in the intention to treat analysis was 28.6% (CI 24.8–32.6) after SG and 31.7% (CI 27.9–35.4) after BSG. Weight-regain from nadir weight was 18.0% EWL (CI 12.8–23.1) after SG and 17.1% EWL (CI 12.3–21.9) following BSG. After initial successful weight loss (EWL > 50%), weight-regain was the indication for revision in 3 patients after SG (mean weight regain of 20.6 kg) and in 1 patient following BSG (weight regain of 25 kg) 5 years after the initial operation. Ten patients following SG and 7 patients after BSG fell below the threshold of poor response (%TWL < 20%).

### Development of Comorbidities and Quality of Life

All patients with T2D (*n* = 17) experienced remission or improvement (Table 2). Mean HbA1c was lower after BSG (BSG 5.5% (CI 5.3–5.8) vs. SG 6.2% (CI 3.3–9.2)). Antihypertensive medication use was stopped in 56.3% after SG and 48% following BSG (Table 2).

**Table 2:** Development of type 2 diabetes and antihypertensive medication use at 5–6 years in relation to baseline, incidence of vitamin deficiencies following banded and non-banded sleeve gastrectomy

Category	Banded sleeve gastrectomy	Sleeve gastrectomy
Type 2 diabetes		
T2D at baseline, no	11	6
Remission, no. (% of base.)	7 (63.6)	4 (66.7)
Improvement, no. (% of base.)	2 (18.2)	1 (16.7)
Unchanged, no. (% of base.)	0	0
Worsened, no. (% of base.)	0	0
HbA1c at baseline, mean (CI)	6.9 (5.6–8.3)	7.1 (5.2–9.1)
HbA1c 5 y, mean (CI)	5.5 (5.3–5.8)	6.2 (3.3–9.2)
Missing at follow-up, no	2	1
Antihypertensive medication use		
Medication use at baseline	25	16
Stopped, no. (% of base.)	12 (48)	9 (56.3)
Lowered, no. (% of base.)	8 (32)	4 (25)
Unchanged, no. (% of base.)	2 (8)	1 (6.3)
Increased, no. (% of base.)	0	1 (6.3)
Missing at follow-up, no	3	1
Vitamin deficiency (ref. interval)		
Vitamin D3 *(50*–*174.7 nmol/l)*		
5 years, no. (% of n)	14 (41.2; *n* = 34)	14 (46.7; *n* = 30)
Vitamin B1 *(89*–*225.3 nmol/l)*		
5 years, no. (% of n)	0 (*n* = 33)	1 (3.4; *n* = 29)
Vitamin B12 *(145.3*–*568.8 pmol/l)*		
5 years, no. (% of n)	1 (2.9; *n* = 34)	0 (*n* = 30)
Folic acid *(10.4*–*42.4 nmol/l)*		
5 years, no. (% of n)	12 (35.3; *n* = 34)	12 (40; *n* = 30)
PTH (1.6–6.9 pmol/l)		
5 years, no. (% of n)	7 (21.2; *n* = 33)	4 (13.8; *n* = 29)
Iron (Ferritin; 31.7–316.5 pmol/l)		
5 years, no. (% of n)	9 (26.5; *n* = 34)	2 (6.5; *n* = 31)

Nineteen (48.7%) patients after SG and 25 (61.0%) following BSG reported no reflux associated symptoms. Eleven patients in either group (SG 28.2%, BSG 26.8%) experienced reflux symptoms $\ge$ 1 per week. The reflux scores GERD HRQL (SG 6.9 (5.1–8.7) vs. BSG 6.4 (4.2–8.5)) and RSI (SG 5.1 (3.4–6.8) vs. BSG 4.7 (2.6–6.8)) were similar in both groups.

Regurgitation was reported by 59% of patients following BSG and by 23% after SG. However, the proportion of patients experiencing frequent regurgitation $\ge$ 1/week was considerably lower with a smaller difference (SG 15.4%, BSG 22.0%). There was no revision due to regurgitation following BSG. BAROS score as a measure of quality of life was comparable in both groups (SG 4.03 (CI 3.34–4.71) vs. BSG 5.59 (CI 3.99–5.29)). Vitamin D and folic acid deficiencies were present in a large proportion of patients (Table 2). Iron deficiency was approximately 4 times higher in patients after BSG (Table 2).

### Long-Term Complications

Type and frequency of minor late complications are described in Table 3. One case of ring slippage was described earlier [10]. Six patients in the SG group and 5 patients after BSG were revised due to clinically relevant reflux symptoms. Of these, 10 patients were converted to an RYGB or BRYGB. In one patient, a proximal re-sleeve was performed as an individual approach to salvage sleeve anatomy. Interestingly, one patient of each group was revised due to dumping syndrome. Both patients had been converted from a sleeve to an RYGB about 2 and a half years earlier.

**Table 3:** Late complications following banded and non-banded sleeve gastrectomy

Complication type and category	Banded sleeve gastrectomy (*n* = 42)	Sleeve gastrectomy (*n* = 40)
Minor late (> 30 d), no. (%)		
Regurgitation $\ge$ 1/ week	9 (21)	6 (15)
Gastroesophageal reflux RSI > 13	4 (10)	3 (8)
Sleeve stenosis	0	1 (3)
Symptomatic cholelithiasis	3 (7)	2 (5)
Total	16 (38)	12 (30)
Major late (> 30 d), no. (%)		
Ring slippage	1 (2)	0
Gastroesophageal reflux with conversion to RYGB	4 (10)	6 (15)
Gastroesophageal reflux with re-sleeve	1 (2)	0
Incisional hernia	0	1 (3)
Internal hernia with operative revision	0	1 pt. with RYGB (3)
Dumping syndrome with operative revision	1 pt. with RYGB (2)	1 pt. with RYGB (3)
Total	7 (17)	9 (22)

### Body Contouring Surgery

BCS was performed in 16 (40.0%) patients after SG and in 17 (40.5%) following BSG. Abdominoplasty was the predominant procedure in both groups (SG *n* = 13, BSG *n* = 17) followed by leg (SG *n* = 9, BSG *n* = 6) and arm (SG *n* = 4, BSG *n* = 3) lifts. Reconstructive breast surgery was performed in 5 patients in each group. Of patients opting for BCS, 50% (*n* = 8) after SG and 41% (*n* = 7) after BSG received more than one type of BSC.

## Discussion

The 5- to 6-year follow-up report of an RCT cohort comparing BSG to SG revealed an EWL difference of 6.3–9.0%. Surgical complications, proportion of T2D resolution, reduction in antihypertensive medication, clinical reflux signs, and quality of life were similar in both groups. The main added morbidity of BSG was regurgitation.

So far, all studies comparing SG to BSG with available mid-term outcome reported superior weight loss for BSG [7–9, 15]. Retrospective analyses of the author’s own group demonstrated an EWL advantage for BSG of 10.8% after 3 and 14% after 5 years [7, 8]. These findings could be confirmed in our group’s RCT, showing a weight loss difference of 11.3% 3 years after surgery [10]. In the current analysis, the weight loss difference was 6.3–9.0% EWL favoring BSG, indicating a continued benefit of ring placement in the RCT cohort 5–6 years after surgery. The gap between intention-to-treat and per-protocol analysis was primarily due to a better weight loss in revised patients of the SG group. However, surgical revisions cannot account for the absence of a progressive weight loss delta with longer follow-up as could have been expected based on retrospective findings. Furthermore, weight regain and number of revisions due to impaired weight loss were similar in the current report. This stands in contrast to the initial hypothesis of less weight regain when banding a sleeve, which has so far been supported by two independent trails [9, 10].

Overall, it is difficult to interpret the clinical relevance of the weight loss difference observed in this report. It is unclear which magnitude of weight loss difference associates with quantifiable clinical or subjective benefits. As a reference, the estimated difference between SG and BSG in this study is comparable with the estimated weight loss delta of a recent pooled analysis of the two largest RCTs comparing SG with RYGB (SM-BOSS and SLEEVEPASS) [16].

An increased amount of postprandial regurgitation was the main added morbidity by banding the sleeve in this report. This finding is consistent with previous studies of the author´s group [7, 8]. However, these retrospective studies reported a rate of frequent regurgitation between 37–44% and a ring removal rate due to regurgitation of 9.8% [7, 8]. This incidence approximately halved in the RCT cohort at 3 and 5–6 years [10]. This reduction is probably associated with the larger implant diameter (2.4 cm vs. 2.0 cm) and a more proximal ring position (4 cm vs. 6 cm) used in this population. Interestingly, most other studies on BSG do not report a larger amount of regurgitation [9, 11, 15]. With respect to the clinical impact of increased postprandial regurgitation in BSG, quality of life was similar in both groups in the current follow-up. In clinical practice, ring removal or enlargement is offered in case of frequent regurgitation. On an individual level, patients often decline ring removal despite frequent regurgitation as they fear weight regain. This may explain the gap in this report between 22% of frequent regurgitation but no subsequent revisions due to this cause.

Ring slippage is a serious ring-related complication and requires immediate ring removal as it may lead to complete obstruction or impairment of the sleeves’ blood supply. Slippage occurred in one patient in this report. Several retrospective trials note one event of ring slippage in one patient suggesting a low incidence of this complication [7–9, 15]. To this date, intraluminal ring migration has only been reported in one case following banded sleeve gastrectomy, yet the incidence of this complication was 1.6% in a large series of almost 3000 patients after banded Roux-en-Y gastric bypass [17, 18].

Although regurgitation is a key feature of GERD, the proportion of patients experiencing reflux symptoms in the current report was comparable in both groups [19]. GERD related regurgitation is defined as “effortless return of gastric contents” by the American College of Gastroenterology guidelines, whereas postprandial regurgitation after ring placement is perceived as immediate and convulsive [19]. Therefore, patients are usually able to differentiate between GERD and ring-related regurgitation. Nevertheless, there may be an overlap in this trial, as GERD was defined clinically without the use of pH probes.

In our RCT comparing SG to BSG, BSG patients experienced significantly less reflux symptoms 3 years after surgery provoking a discussion on silicone ring placement as a potential anti-reflux procedure [10]. However, neither endoscopic findings at 3 years nor the incidence of reflux symptoms in the current report can support an anti-reflux effect of ring placement [10].

A large US cohort study demonstrated that 5.6% of patients had received BCS at least 4 years following bariatric surgery [20]. The group attributed this, among other reasons, to the high costs of BCS [20]. In Austria, where insurance companies typically cover BCS in presence of a medical indication, the rate of BCS at least 2 years following gastric bypass was 14.9% [21]. The proportion of patients having received BCS in the current study is more than twice as high (40%). In Germany, where insurance companies typically also cover BCS similarly to Austria, this could possibly be explained with the high baseline BMIs found in this cohort in comparison to international thresholds.

The current analysis is a follow-up report based on an RCT cohort. It was not powered to compare clinical outcome differences at 5–6 years. Furthermore, drop-outs may lead to unequal distribution of confounding variables. This limitation mainly concerns weight loss as it was the primary endpoint of the RCT. Therefore, the statistical analysis is descriptive. In the current 5- to 6-year follow-up, population size decreased by 29% in the per-protocol population. Sample size calculations of the RCT were powered to detect a treatment difference of 8% EWL, assuming a drop-out rate of 10%. Consequently, the current analysis was underpowered to detect weight loss differences in that range.

## Conclusion

This 5-year follow-up report of an RCT comparing banded to non-banded sleeve gastrectomy shows durable weight loss, a high remission rate of type 2 diabetes, as well as a large decrease in antihypertensive medication use in both trial groups. Revision rates ranged between 14.3% for BSG and 20% following SG with revisions predominantly performed due to symptomatic reflux. Patients after BSG had a weight loss advantage of 6.3–9% EWL. This group experienced a high rate of regurgitation as the main added morbidity.
